# Oculomotor Performances Are Associated With Motor and Non-motor Symptoms in Parkinson's Disease

**DOI:** 10.3389/fneur.2018.00960

**Published:** 2018-11-28

**Authors:** Yu Zhang, Aijuan Yan, Bingyu Liu, Ying Wan, Yuchen Zhao, Ying Liu, Jiangxiu Tan, Lu Song, Yong Gu, Zhenguo Liu

**Affiliations:** ^1^Department of Neurology, Xinhua Hospital Affiliated to Shanghai Jiao Tong University School of Medicine, Shanghai, China; ^2^Key Laboratory of Primate Neurobiology, CAS Center for Excellence in Brain Science and Intelligence Technology, Institute of Neuroscience, Chinese Academy of Sciences, Shanghai, China

**Keywords:** oculomotor performance, saccade latency, Parkinson's disease, pursuit gain, Chinese

## Abstract

**Background:** Parkinson's disease (PD) patients exhibit deficits in oculomotor behavior, yet the results are inconsistent across studies. In addition, how these results are associated with clinical symptoms is unclear, especially in China.

**Methods:** We designed a case-control study in China including 37 PD patients and 39 controls. Clinical manifestations in PD patients were recorded. Oculomotor performance was measured by a video-based eye tracker system.

**Results:** We found that six oculomotor parameters, including fixation stability, saccadic latency, smooth pursuit gain, saccade frequency, viewing range, and saccade frequency during free-viewing context, were significantly different in PD patients and control group. Combining application of these six parameters could improve diagnostic accuracy to over 90%. Moreover, pursuit gain was significantly associated with PD duration, UPDRS III, in PD patients. Saccade latency was significantly associated with PD duration, Berg balance score, RBD score, and Total LEDD in PD patients.

**Conclusions:** PD patients commonly exhibit oculomotor deficits in multiple behavioral contexts, which are associated with both motor and non-motor symptoms. Oculomotor test may provide a valuable tool for the clinical assessment of PD.

## Introduction

Parkinson's disease (PD) is a common neurodegenerative disease characterized by the death of dopaminergic neurons in the substantia nigra. Although the pathogenic mechanism of PD remains elusive, it is commonly accepted that genetic components, inflammation, and aging are highly correlated with the occurrence of the disease (1). Parkinson's disease usually causes multiple symptoms including both progressive motor symptoms, such as bradykinesia, rigidity, and tremor, and non-motor symptoms, such as cognitive impairments and mood disorders (2). Growing evidence indicates that several biomarkers might serve as a valuable tool for the diagnosis of parkinson's disease, such as combined cystatin C and low-density lipoprotein cholesterol, trefoil factor3, and image feature (3–9).

Oculomotor dysfunction is a common clinical sign of PD, but the tools available for accurate quantification are limited. The rapid development of the non-invasive, infrared eye tracker systems with high spatial and temporal resolutions, has made it possible to measure precise and accurate eye movements under computer-controlled behavioral paradigms, in both clinical and laboratory conditions. Several studies have indicated that PD patients exhibit deficits in oculomotor behavior compared to the normal subjects, such as saccade and smooth pursuit eye movements (10–15). Although the underlying neural mechanisms remain unclear, it is possible that PD is a multisystem disorder involving multiple brain regions and pathways that are related to eye movements and attention.

Although oculomotor performance in PD has been reported, the results are not always consistent. In addition, whether these oculomotor deficits are associated with clinical diagnosis, including motor and non-motor symptoms, is not clear. Therefore, in the current study our aim is to (1) analyze the oculomotor performance in PD patients using infrared eye tracker, and (2) study the association between oculomotor performance and the motor and non-motor symptoms in PD patients in China.

## Materials and methods

### Subjects

Thirty-seven PD patients were recruited from the Department of Neurology, Xin Hua Hospital, affiliated to Shanghai Jiao tong University School of Medicine. Patients with PD were diagnosed based on the criteria of Movement Disorder of Society, and all the patients received dopaminergic treatment (2, 18). For the control, 39 healthy age- and gender-matched subjects were recruited. Exclusion criteria included previous history of other neurological or psychological conditions, such as stroke, moderate or severe head injury, major depression, brain tumor, learning disability, a history of cranial neurosurgery, major heart disease, and cataract.

### Clinical assessment

All participants received a clinical questionnaire and a systematic medical history record which included details such as age of PD onset, duration of disease, predominant symptoms of PD onset, and complications. The participants were also made to undergo a complete neurological examination. PD patients were assessed using the Hoehn and Yahr modified staging scale (H-Y), Unified Parkinson's Disease Rating Scale (UPDRS), the scale for freezing of gait, Minimum Mental State Examination (MMSE), Non-Motor Symptom (NMS) assessment scale, REM Sleep Behavior Disorder Questionnaire Hong Kong (RBDQ-HK), Hamilton Anxiety Scale (HAMA), Hamilton Depression Scale (HAMD), Parkinson Disease Sleep Scale (PDSS), Parkinson's Disease Questionaire-39 (PDQ-39), and Berg balance scale. All the patients were tested at “on” stage.

### Oculomotor recordings

**Figure 1:**
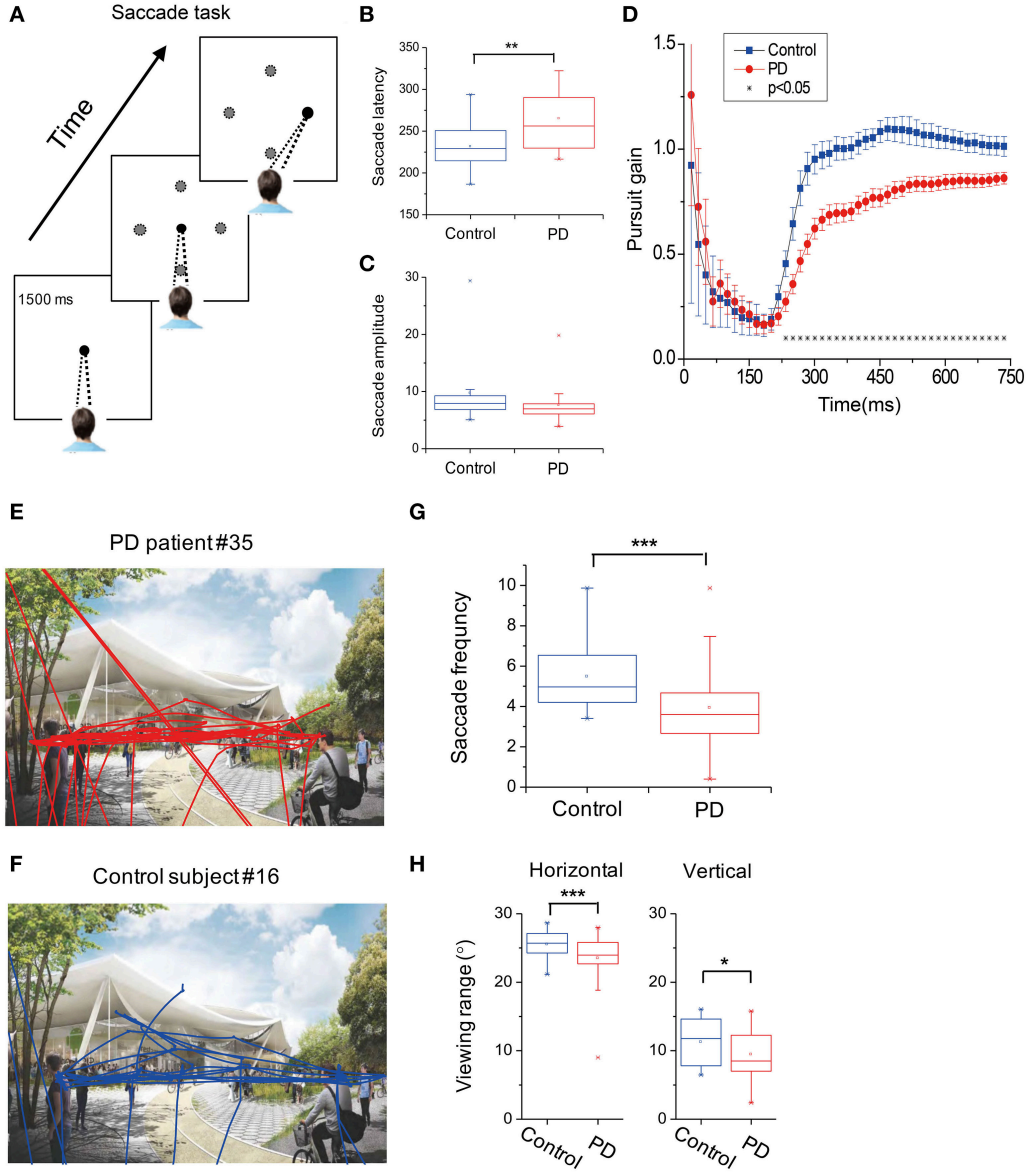
Oculomotor recordings of subjects. **(A)** Subjects were required to fixate a central fixation point for 1,500 ms. After that, the fixation point disappeared and a peripheral target appeared at one (black circle) of the four possible locations (gray circles) along the horizontal and vertical cardinal axes. Subjects were required to saccade to the peripheral target as soon as possible. **(B,C)** Averaged saccade latency **(B)** and saccade amplitude **(C)** between PD and control groups. **(D)** Subjects were instructed to pursue a moving target at a constant speed across the screen. Pursuit gain was defined as the relative distance between the PD patients (red symbols) and normal subjects (blue symbols) along the pursuit axis. Error bars are SEM. Asterisks represent cases with significant difference between the two groups. **(E,F)** Example of eye traces in one PD patient (upper panel) vs. one normal subject (lower panel). **(G,H)** Summary of the saccade eye movements **(G)** and viewing range **(H)** of PD (red symbols) and control (blue symbols) groups. ^*^p < 0.05; ^**^p < 0.01; ^***^p < 0.001.

Eye movements were recorded with a video-based eye tracker (EyeLink 1000, Canada)—an infrared pupil and corneal reflection tracking system which acquired monocular samples at 500 Hz. One computer controlled the eye tracking system while another computer presented the stimuli using custom software and the open source presentation program, PsychoPy. The subject was seated, with his or her head rested on the height-adjustable chin-rest of the eye tracker. A 19^′′^ LCD monitor was placed at a distance of 60 cm in front of the subject's eyes. The resolution was 1,280 × 1,024 pixels with a vertical refresh rate of 60 Hz. The eye tracker system was calibrated prior to each recording session, using a 9-point grid covering the area in which targets were presented (Figure 1A). The parameters that we used in our tests were similar to those used in previous studies (10, 11, 19, 20).

#### Visually guided saccade task

In saccade task, subjects were required to fixate a small white target (0.5° in radius) in the center of the screen for 1 s. At the end of the trial, the central fixation point disappeared and another peripheral white target appeared at an eccentric position of 7°. The saccade target appeared in one of the four directions (up, down, left, and right) and was randomized across trials. Subjects were required to make a saccade to the peripheral target as soon as the fixation point disappeared (Figure 1A). The task was repeated 2 times, with a total of 4 × 2 = 8 trials for each subject.

#### Smooth pursuit task

In the smooth pursuit task, subjects were required to fixate at a small white target (0.5° in radius) for 1.5 s. The target first appeared at one of the four eccentric positions of 7.5° along the horizontal or vertical median (azimuth and elevation: [+7.5, 0°], [−7.5, 0°], [0°, +7.5°], [0°, −7.5°]). After the fixation for 1.5 s, the target started to move with a constant speed of 20°/s along the horizontal or vertical axis. The average eye position during a pursuit trial was aligned with the center of the screen. The subjects were required to pursue the target for 0.75 s. Each condition was repeated 2 times, such that the total number of trials for each subject was 4 × 2 = 8 trials. Different conditions were interleaved within each session. The overall experimental paradigm was similar to that used in previous studies but with some variations (10, 11, 19, 20).

#### Freely viewing task

In the freely viewing Task, a landscape picture was displayed at the center of the screen for 15 s. The image was subtended at a visual angle of 30° × 15° from the subjects' perspective. Subjects were required to freely explore the picture within the image range.

### Statistical analysis

Statistical analysis was performed using SPSS (IBM), OriginPro (OriginLab Corporation), and MATLAB (Mathworks). In all statistical tests, the level of significance was set at *p* < 0.05. Odds ratio (OR) and 95% confidence intervals (95% CI) are presented.

#### Comparison of oculomotor parameters between groups

The mean variables between two groups were compared using a parametric test such as Pearson's Chi-Square test and Student's *t-*test; multiple comparison analysis testing in ANOVA was used when multiple groups of data were involved. We also applied the Receiver operating characteristics (ROC) analysis, a non-parametric test, to quantify the separation of two distributions. A binary logistic regression model was used when multiple parameters were involved before applying the ROC analysis (SPSS, IBM).

#### Principle component analysis of multiple oculomotor variables

The principle component analysis (PCA, MATLAB) was applied to analyze the variables that account for the most variance in the data. Clustering analysis was also applied to automatically group data in a two-dimensional space by using K-means (MATLAB).

#### Correlation between oculomotor performance and clinical diagnosis

The relationship between two oculomotor parameters was assessed using non-parametric Spearman rank correlation. A 95% confidence interval was computed for the correlation coefficient (OriginPro, OriginLab Corporation).

## Results

### Demographic and clinical of subjects

Demographic and Clinical of Subjects information of our patients and control are listed in Table 1. There was no significant difference in age and gender distribution among the two groups. The duration of disease was 5.61 ± 0.89 years. The mean UPDRS III motor score for the entire study group was 14.76 ± 1.54. Among the PD patients, 31 patients (83.78%) were in the early stages of PD (H & Y stage 1–2) and 6 (16.22%) were in the later stages of PD (H & Y stage 2.5–3. 0) (Table 1).

**Table 1:** Demographic and clinical characteristics of PD patients and control.

**Characteristic**	**Control (*n* = 39)**	**PD (*n* = 37)**	***P***
Men, No, (%)	21(53.85%)	23 (62.16%)	0.585
Age (years)	67.18 ± 1.20	67.14 ± 0.99	0.978
PD duration		5.61 ± 0.89
UPDRS III		14.76 ± 1.54
**HOEHN-YAHR**			
1–2		31 (83.78%)
2.5–3		6 (16.22%)

*UPDRS, Unified Parkinson's disease Rating Scale*.

### Oculomotor performance

#### Fixation stability

Static gaze stability was assessed through the standard error of the average eye position during the fixation period (1.5 s). Fixation stability was significantly worse in PD patients compared to the control (*p* = 0.0097, *t-*test).

#### Saccade eye movements

In the visually guided saccade task (Figure 1A), the average saccadic latency to the peripheral target was significantly increased by 16.3% (*p* = 0.0011, *t-*test) in PD patients (258.9 ± 5.3 ms) in comparison to the control subjects (228.1 ± 4.1 ms, Figure 1B). As for the saccade amplitude, PD patients showed smaller eye movement distance compared to the control; however, it did not reach a statistical significance level (*p* = 0.053, *t-*test, Figure 1C).

#### Smooth pursuit eye movements

In the smooth eye movement task, we calculated the gain between the eye position and the visual target position along the pursuit direction at each time point. A gain of 1 indicates that the actual eye movement is perfectly aligned with the moving target. A gain of < 1 indicates that there is a lag between the eye movements and the visual target, and a gain of >1 indicates overshooting. For both groups of subjects, the pursuit gain was smaller than 1 during the initial period when the target started moving (Figure 1D). However, after a few hundredths of a millisecond, the normal control subjects gradually caught the target (i.e., gain ≈ 1), whereas the PD patients also gradually caught the target but with some lag (i.e., gain < 1). This difference in pursuit gain between the groups was statistically significant at around 200 ms after the target motion and then remained consistent throughout the whole trial (*p* < 0.05, *t-*test, indicated by asterisks in Figure 1D). This phenomenon was similar in both the horizontal and vertical axes, without a statistically significant difference (*p* > 0.05, Two-way ANOVA).

#### Free-viewing context

In the free-viewing context, subjects were allowed to look anywhere within the presented landscape picture for 15 s (Figures 1E,F). We found that the PD patients tended to make less saccadic eye movements compared to the normal subjects (Figure 1G, *p* = 7.8E-4, *t-*test). Moreover, compared to the control group, PD patients tended to view in a narrower range, particularly in the horizontal direction (*p* = 0.0069, *t-*test, Figure 1H, left panel), and this trend also happened in the vertical direction but with a relatively weaker effect (*p* = 0.045, *t-*test, Figure 1H, right panel).

### Correlation between oculomotor performance and clinical variables

#### Receiver operating characteristics (ROC) analysis

**Figure 2:**
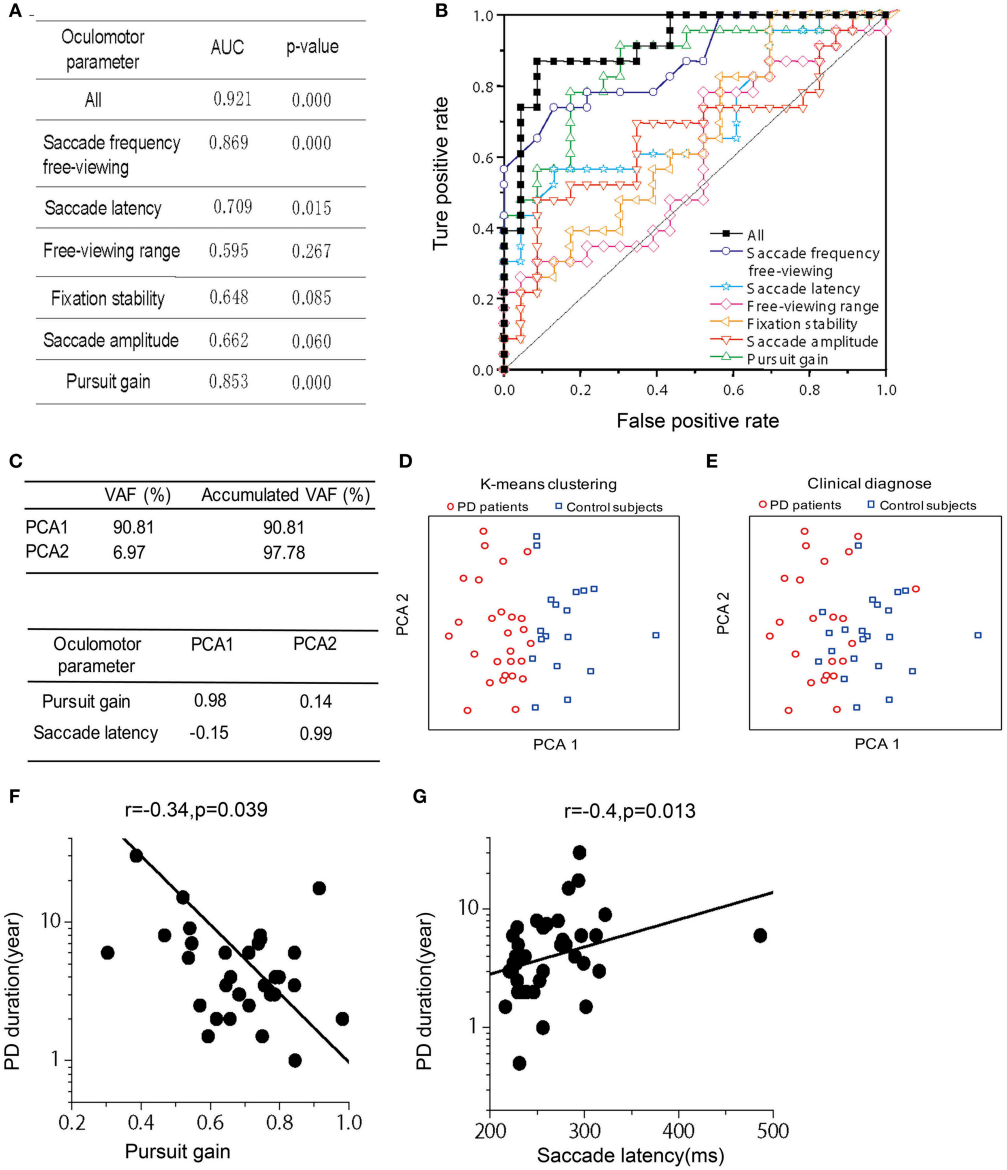
Receiver operating characteristics (ROC) analysis, Principle Component Analysis (PCA), and Correlation between PD duration and oculomotor parameters of pursuit gain and saccade latency. **(A)** Area under the curve (AUC) for the six oculomotor parameters. Unity line indicates 50% chance of the hit and false alarm rate. The combined (black-filled symbols) AUC was acquired based on all the six parameters through a logistic fit. **(B)** The AUC- and *p*-value for each and the combined oculomotor parameters. **(C)** Upper box shows the accumulated variance accounted for (VAF) by the first two principle components. Lower box shows correlation coefficients of the oculomotor parameters in the first two principle components. **(D)** The first two principle components were plotted against each other, with red and blue symbols indicating two clusters defined from a k-means clustering method. **(E)** The first two principle components were plotted against each other, with red and blue symbols indicating PD patient and normal group, respectively, as diagnosed according to clinical criteria. **(F,G)**. Correlation was assessed through Spearman rank correlation. The fitted line was from type II linear regression by minimizing the orthogonal distance between the data and the fitted line.

We used the non-parametric ROC analysis to test an ideal observer's performance of identifying PD vs. control groups based on the above-mentioned six oculomotor parameters, which include (1) saccade frequency during the free-viewing context, (2) saccade latency, (3) viewing range during the free-viewing context, (4) fixation stability, (5) saccade amplitude, and (6) pursuit gain. Among them, pursuit gain, saccade latency, and saccade frequency during free-viewing exhibited highest correct rate (AUC: 0.853, 0.709, and 0.869; *p*-value: 0.000, 0.015, and 0.000, respectively; Figure 2A). We also used ROC analysis to perform a combined diagnosis by considering all the six oculomotor parameters. We found that the ideal observer's correct rate could be increased to 92.1% (Figures 2A,B, black symbols). This result suggests that the oculomotor performance could largely separate the two groups of subjects.

#### Principle component analysis (PCA)

In addition to the ROC analysis, we further performed principle component analysis (PCA) by including the same 6 oculomotor parameters as described in the above section. First, we found that the first two principle components among the high-dimensional space accounted for a majority of the total variance in the data (VAF: ~98%). It is important to note that these two principle components were mainly contributed by pursuit gain and saccade latency, respectively (Figure 2C). Second, as shown in Figure 2D, the data were spread in a two-dimensional space based on the first two principle components. Data were partitioned into two groups through a K-means clustering method. Interestingly, these two clusters roughly corresponded to the two groups of PD and control as diagnosed based on the clinical criteria (Figure 2E).

### Correlation of abnormal oculomotor performance and clinical characteristics in PD patients

Since the PCA showed that pursuit gain and saccade latency were the major contributors in identifying PD vs. control (explained ~98% of variance in the data), we further examined whether these two oculomotor parameters were significantly correlated with the clinically evaluated variables of the PD patients. We found that pursuit gain significantly correlated with PD duration (*r* = −0.337, *p* = 0.039, with a 95% CI of −0.576~−0.014, Spearman rank correlation, Figure 2F) and UPDRS scores, especially UPDRS III (*p* = 0.046, Table 2). As for saccade latency, it significantly correlated with PD duration (*r* = 0.406, *p* = 0.013, with a 95% CI of −0.094~−0.645, Spearman rank correlation, Figure 2G), RBDSQ-HK scores (*r* = 0.604, *p* = 1.2E-4, with a 95% CI of −0.601~−0.002, Spearman rank correlation, Table 2), and Berg Balance Scale (*r* = −0.335, *p* = 0.043, with a 95% CI of 0.339~0.780, Spearman rank correlation, Table 2). Thus, the only clinical variable that significantly correlated with both the oculomotor parameters was the disease duration. Specifically, patients with longer disease duration tended to exhibit a smaller pursuit gain (Figure 2F) and a greater saccadic lag (Figure 2G).

**Table 2:** Significance of the correlation between clinical evaluations and oculomotor performance of pursuit gain and saccade latency.

	**Pursuit gain**			**Saccade latency**		
	***p***	***r***	**95% CI**	***p***	***r***	**95% CI**
Gender	0.759	0.103	−0.229~0.413	0.489	0.048	−0.280~0.367
Age	0.399	−0.140	−0.444~0.193	0.193	0.221	−0.111~0.509
PD duration	0.039^*^	−0.337	−0.576~-0.014	0.013^*^	0.406	0.094~0.645
Rigidity/tremor	0.532	0.246	−0.085~0.528	0.232	0.325	0.001~0.587
UPDRS total	0.356	−0.264	−0.610~0.166	0.218	0.269	−0.161~0.613
UPDRS I	0.856	0.067	−0.355~0.466	0.422	0.199	−0.232~0.565
UPDRS II	0.732	−0.070	−0.468~0.353	0.813	0.058	−0.363~0.459
UPDRS III	0.047^*^	−0.396	−0.684~-0.001	0.149	0.301	−0.107~0.622
UPDRS IV	0.860	0.256	−0.174~0.605	0.160	0.482	0.087~0.746
Hoehn-Yahr stage	0.820	0.011	−0.323~0.343	0.443	0.177	−0.167~0.481
Freezing of gait	0.191	−0.152	−0.462~0.191	0.863	0.089	−0.252~0.410
Berg Balance Scale	0.974	0.012	−0.322~0.344	0.043^*^	−0.335	−0.601~−0.002
MMSE score	0.929	0.030	−0.306~0.360	0.916	−0.004	−0.336~0.330
HAMA score	0.763	−0.048	−0.375~0.290	0.995	0.006	−0.328~0.338
HAMD score	0.884	0.028	−0.308~0.358	0.356	−0.158	−0.467~0.185
RBDSQ-HK score	0.539	−0.103	−0.422~0.238	0.000127^***^	0.604	0.339~0.780
PDSS	0.774	−0.050	−0.377~0.289	0.784	−0.047	−0.375~0.291
NMS score	0.083	−0.308	−0.593~0.046	0.135	0.272	−0.084~0.567
PDQ-39 score	0.819	−0.037	−0.381~0.316	0.807	0.050	−0.304~0.392
Total LEDD	0.074	−0.298	−0.567~0.029	0.010^*^	0.417	0.107~0.653
L-dopa LEDD	0.270	−0.186	−0.481~0.147	0.002^**^	0.497	0.206~0.707
Non-L-dopa LEDD	0.068	−0.303	−0.571~0.023	0.023^*^	0.373	0.056~0.622

*P-values were acquired from non-parametric test of the Spearman rank correlation*.

*UPDRS, Unified Parkinson's Disease Rating Scale; MMSE, Minimum Mental State Examination; HAMA, Hamilton Anxiety Scale; HAMD, Hamilton Depression Scale; RBDSQ-HK, REM Sleep Behavior Disorder Questionnaire Hong Kong; PDSS, Parkinson Disease Sleep Scale; NMS, Non-Motor Symptom Assessment Scale; PDQ-39, Parkinson's Disease Questionaire-39*.

* *p < 0.05*,

** p < 0.01, and

*** *p < 0.001*.

## Discussion

Oculomotor recordings have been applied in many brain-related diseases such as Schizophrenia (21), Alzheimer's disease (22), Autism (23), and Huntington's disease (24). One important reason for their application is that the fast-developing eye tracker system, based on non-invasive video graph, could conveniently measure the dynamic eye positions and status by providing a much more accurate, and thorough (e.g., pupil size) information that cannot be easily captured by traditional methods such as electrooculogram (10). Meanwhile, impaired oculomotor behavior in PD has long been recognized, including both saccade and smooth pursuit eye movements, although there were several conflicting reports across different studies from different laboratories, probably due to different experimental conditions (10–12, 16, 17, 20).

In the current study, we have also observed abnormal oculomotor performance in PD patients. In particular, the significant difference in the eye movements of the control subjects includes less fixation stability, longer saccade latency, smaller smooth pursuit gain, and less saccade eye movements and narrower viewing range during free-viewing. In addition, we found that these oculomotor alterations in PD are significantly associated with several clinical variables related to motor and non-motor symptoms, including PD duration, UPDRS III, Berg balance score, and RBD score. In particular, both pursuit gain and saccade latency are significantly correlated with PD duration. Therefore, our results indicate that measuring of eye movements could be a potentially useful tool for assessing the disease.

Compared with previous studies, there are a number of consistent results. Deficits in saccade latency and pursuit gain have been observed in most of the previous studies (11, 12). In addition, Buhmann's study reported a 25% decreased rate of successful visual searches in PD patients (14). This is consistent with our results where PD patients tend to have less saccadic eye movements and narrower viewing range during a free-viewing context. In another study, Ewencyzk and colleagues showed that the impairment of anti-saccade latencies significantly correlated with impaired release of anticipatory postural program (15). Although we do not have an anti-saccade task in our current study, our PD patients exhibited impairment in saccade latency in the visually guided saccade task, and this impairment significantly correlated with a low Berg balance score. Therefore, the delay of saccade latency may be helpful in predicting the risk of falling. Meanwhile, we found that saccade latency in PD patients was also associated with the RBD score, Suggesting that both of autonomous and non-autonomous eye movements of PD patients are affected. As most of our patients are in the early-stages of PD, and RBD has been thought to be an effective tool for the early diagnosis of PD, the fact that saccade latency is associated with RBD suggests that saccade latency may be important for early diagnosis of PD. On the other hand, our results indicated that saccade latency is associated with the use of dopamine medicine, which is consistent with previous studies (25, 26). These findings suggest that levodopa improves the function of the voluntary frontostriatal system, which is deficient in PD.

However, there are also several limitations in our study. First, although statistics and ROC analysis revealed significant differences between the patients and the normal subjects on the population level, the overlap between the two groups limits the application of the oculomotor datafor individual clinical diagnosis. Thus, at least two strategies could be used in future tests. One strategy is to design more efficient tasks to improve the discriminability between the patients and control groups. The other strategy, however, is to improve the clustering efficiency of the PCA. Note that the classification defined based on the objective PCA and that based on the clinical criteria in hospitals may not have to be identical. Thus, their relationships need to be further explored in the future. Second, the parameters of oculomotor performance may be affected by other non-PD-specific factors such as fatigue, because PD patients tend to fatigue when they participate in a series of assessments. Deficits in oculomotor behavior have also been observed in other diseases such as Schizophrenia, Alzheimer's disease, Autism, and Huntington's disease. Thus, we require better experimental design and tests in future studies to identify PD more efficiently and also to distinguish PD from other diseases. Third, the oculomotor impairments are observed in the early and moderate stages of PD patients who have been identified based on clinical diagnosis in hospitals and received dopamine medicine therapy. We have not measured the oculomotor behavior in pre-symptomatic subjects or PD patients without dopamine medicine therapy. In the future, oculomotor behavior needs to be repeatedly measured in *de novo* PD population across a long life span, which may provide more interesting and useful information.

In summary, our study confirmed abnormal oculomotor behavior in PD and demonstrated remarkable correlations with several motor and non-motor symptoms in PD patients. Our findings suggest that oculomotor examination may serve as a valuable means to assist in the clinical assessment of Patients with PD. In future, studies are particularly required to assess the value of eye movement tracking for differentiating between PD and other movement-related diseases.

## Ethics statement

Informed written consent was obtained from all subjects. This study was performed with the approval of the Ethics Committee of Xin Hua Hospital affiliated to Shanghai Jiao tong University School of Medicine (XHEC-C-2016-150-19).

## Author contributions

YZ, YG, and ZL conceived the project and designed the study. AY, BL, YcZ, YL, JT, YW, and LS contributed to participant recruitment, data collection, and data analysis. YZ, AY, and BL wrote the paper.

### Conflict of interest statement

The authors declare that the research was conducted in the absence of any commercial or financial relationships that could be construed as a potential conflict of interest.
